# A Randomized Clinical Trial Comparing Eyetronix Flicker Glass and Patching for Treatment of Amblyopia in Children Reveals Similar Improvements in Vision

**DOI:** 10.3389/fnins.2021.622729

**Published:** 2021-04-09

**Authors:** Seung Hyun Min, Shijia Chen, Jinling Xu, Bingzhen Chen, Hui Chen, Yuwen Wang, Jiawei Zhou, Xudong Yu

**Affiliations:** ^1^School of Ophthalmology and Optometry and Eye Hospital, State Key Laboratory of Ophthalmology, Optometry and Vision Science, Wenzhou Medical University, Wenzhou, China; ^2^Department of Ophthalmology and Visual Sciences, McGill Vision Research, McGill University, Montreal, QC, Canada

**Keywords:** amblyopia, eyetronix flicker glass, randomized controlled trial, patching, visual acuity

## Abstract

**Purpose:**

Recently, Eyetronix Flicker Glass (EFG) has been introduced as a novel treatment for amblyopia. It alternatively deprives the visual input of each eye rapidly (e.g., 7 Hz). However, whether it is comparable with standard patching therapy is unclear. In this randomized clinical trial, we evaluate the efficacy of an EFG therapy as treatment for amblyopia in children and compare it to the patching therapy.

**Methods:**

We tested 31 children (aged 4–13 years) with amblyopia. They were assigned into one of the two treatment groups and were treated for 12 weeks. The first group was treated with EFG for 1 h/day (Flicker Group) and the latter with a standard patch (Patching Group) for 2 h/day. We designated changes from baseline in best-corrected visual acuity (BCVA) of the amblyopic eye as our primary outcome. Changes from baseline in other visual outcomes, such as contrast sensitivity, stereopsis, and fusional vergence range were measured as secondary outcome.

**Results:**

BCVA improved significantly at 12 weeks relative to baseline in both the Flicker (0.13 ± 0.11 logMAR; mean ± SD) and Patching Groups (0.21 ± 0.14 logMAR). However, the improvements were not significantly different between groups (*p* = 0.13). Contrast sensitivity also significantly improved at 3 and 12 cycles/degree between baseline and 12 weeks in both groups (*p*’s < 0.05). However, stereopsis and fusion range did not improve significantly in both groups.

**Conclusion:**

An EFG therapy and patching improved BCVA similarly for children with amblyopia at 12 weeks. Both therapies improved the contrast sensitivity at 3 and 12 cycles per degree (cpd); however, only patching improved the contrast sensitivity at 6 cpd. Both therapies did not benefit binocular visual functions (stereopsis and fusional vergence range). We believe that EFG can be an additional choice for therapy.

**Clinical Trial Registration:**

chictr.org number: ChiCTR2000034436.

## Introduction

Amblyopia is a neurodevelopmental disorder from abnormal visual experience during the critical period (Hess and Thompson, 2015; Levi et al., 2015). Early treatment is necessary for a proper recovery. In clinics, amblyopic children are prescribed with a patching therapy, which deprives the fellow eye to force the amblyopic eye to work (De Buffon, 1743). Younger children have been reported to benefit more than older counterparts (Fronius et al., 2014). It improves the visual acuity (VA) of the amblyopic eye by more than two lines of logMAR in over 50% of cases (Repka et al., 2003; Scheiman et al., 2005). However, it discomforts the children because only the amblyopic eye is opened (Stewart et al., 2004; Fronius et al., 2014). Alternative therapies, such as atropine penalization and Bangerter filters, have been used, but their premise is the same as patching: the input in the fellow eye is inhibited. These monocular therapies have been shown to bring suboptimal results, poor compliance, and harm to binocular function (Searle et al., 2002; Hess and Thompson, 2013; Wallace et al., 2013; Papageorgiou et al., 2019).

Recently, Eyetronix Flicker Glass (EFG) has been introduced as a novel treatment for amblyopia. The lenses in EFG for both eyes flicker alternatively between opaque (“off”) and transparent (“on”). Hence, EFG therapy differs from the patching therapy because it alternatively deprives each monocular input rapidly (e.g., 7 Hz) and enables the patients to see with both eyes throughout the treatment (Spierer et al., 2010; Wang et al., 2016). Moreover, it differs from binocular therapies with a dichoptic training protocol that have been developed in the last decade. A dichoptic training protocol displays stimuli separately (i.e., dichoptically) and simultaneously to each eye in the form of movie viewing (Li et al., 2015b), video gaming (Knox et al., 2012), and perceptual learning (Vedamurthy et al., 2015). On the other hand, patients do not receive binocular input at any moment during an EFG therapy.

When a new amblyopia treatment is developed, one must resolve whether it produces a comparable VA gain as a standard therapy such as patching. Likewise, dichoptic therapies have been extensively studied in randomized controlled trials (RCTs). While some RCTs in children show that their superiority to patching remains elusive (Manh et al., 2018; Holmes et al., 2019), others show that a novel binocular treatment is superior to patching (Kelly et al., 2016). A recent study from Eyetronix^®^ shows that EFG therapy for 12 weeks improves both the VA of the amblyopic eye and stereopsis (Vera-Diaz et al., 2016). However, it does not compare against a control group of patching. We conducted a randomized clinical trial to compare EFG therapy with patching at a non-profit research hospital. Just as in previous RCTs of binocular therapies (Kelly et al., 2016; Birch et al., 2019), we designated changes from baseline in best-corrected VA (BCVA) as our primary outcome. Moreover, VA of the amblyopic eye has been the primary measure in diagnosing and treating amblyopia (Wallace et al., 2018). Changes from baseline in other visual functions, such as contrast sensitivity, stereopsis, and fusional vergence range, are reported as secondary outcome.

## Materials and Methods

This RCT is listed in the Chinese Clinical Trial Registry^1^ with the identifier ChiCTR2000034436. The full trial can be accessed from the Chinese Clinical Trial Registry’s website: http://www.chictr.org.cn/showproj.aspx?proj=56030. The research protocol and the informed consent forms were reviewed and approved by the Ethics Committee of the Affiliated Eye Hospital at Wenzhou Medical University (2016-18-Q-11). All data were collected at the Affiliated Eye Hospital of Wenzhou Medical University.

### Patients

Thirty-two children (aged 4–13) with untreated, mild-to-severe unilateral amblyopia participated in our study. One of them received another treatment during the study and had to be excluded, so 31 children completed the study. The clinical details of the patients are provided in Table 1. We obtained informed consent from their parent or legal guardians. The sample size was determined based on the previous studies of EFG and liquid crystal glasses (LCG) (Spierer et al., 2010; Vera-Diaz et al., 2016). Their guardians volunteered to participate in the study and accepted the random assignment of each patient to either Group 1 (therapy with the EFG) or Group 2 (traditional patching therapy).

**TABLE 1:** Baseline characteristics of the patients.

	**Flicker group**		**Patching group**	
	**(*N* = 15)**		**(*N* = 16)**	
	***N***	**%**	***N***	**%**
**Gender**				
Female	6	40%	8	50%
**Age at enrollment (years)**				
4 to < 6	9	60%	6	38%
6 to < 8	5	33%	5	31%
8 to ≤13	1	7%	5	31%
Mean ± SD	5.27 ± 1.10		6.38 ± 2.45	
**Distance visual acuity of amblyopic eye (logMAR)**				
0.15 to < 0.2	1	7%	1	6%
0.2 to < 0.3	3	20%	3	19%
0.3 to < 0.4	3	20%	5	31%
0.4 to < 0.5	3	20%	2	13%
0.5 to < 0.6	3	20%	2	13%
0.6 to < 0.7	1	7%	0	0%
0.7	1	7%	3	19%
Mean ± SD	0.39 ± 0.16		0.38 ± 0.19	
**Distance visual acuity of fellow eye (logMAR)**				
Mean ± SD	0.03 ± 0.05		0.02 ± 0.09	
**Interocular acuity difference (logMAR)**				
Mean ± SD	0.37 ± 0.19		0.37 ± 0.17	
**Baseline stereoacuity (arcseconds)**				
Nil (converted to 4,500)	4	27%	6	40%
800	0	0%	1	7%
400	3	20%	2	13%
200	2	13%	0	0%
140	0	0%	2	13%
100	1	7%	0	0%
80	0	0%	3	20%
60	1	7%	1	7%
50	2	13%	0	0%
40	2	13%	1	7%
Mean ± SD (arcseconds)	1,329.33 ± 1,983.41		1,826.25 ± 2,147.26	
Mean ± SD (log arcseconds)	2.49 ± 0.81		2.71 ± 0.82	
**Amblyopic eye’s spherical equivalent (diopters)**				
Mean ± SD	3.95 ± 2.58		4.05 ± 1.76	
**Fellow eye’s spherical equivalent (diopters)**				
Mean ± SD	0.85 ± 1.50		0.77 ± 1.41	
**Squint (Δ) by PACT**				
0	8	53%	13	81%
1 to < 10	6	40%	12	13%
10 to < 20	1	7%	1	6%
Mean ± SD (Δ)	−2.40 ± 3.87		−1.63 ± 4.21	

*logMAR, logarithm of the minimum angle of resolution; SD, standard deviation; PACT, Prism and Alternate Cover Test.*

We estimated the sample size via power analysis (Hickey et al., 2018). To do so, we extracted from the literature a typical improvement of the amblyopic eye’s VA in amblyopic children after patching. Studies show that patching for 12 weeks improves VA by about 0.21–0.35 logMAR (Stewart et al., 2004; Rutstein et al., 2010), which were then averaged for our analysis (i.e., 0.273 logMAR). Also, treatment using flickering goggles improves it by 0.124 logMAR (Vera-Diaz et al., 2014; Vera-Diaz et al., 2016). With a power of 80%, an α level of 0.05, a difference (i.e., absolute effect size) of 0.149 logMAR between the improvements in VA shown by the two treatment methods, and a standard deviation of 0.11 logMAR (Rutstein et al., 2010; Rajavi et al., 2019), we found that the minimum sample size per group would have to be nine patients. To make our study robust, we recruited 16 children for each treatment group, although one of them later withdrew from the study.

*Eligibility inclusion criteria:* (1) age range of 4 to 13 years; (2) a diagnosis of amblyopia, BCVA of the amblyopic eye equal to or worse than 0.3 (logMAR) in 3- to 5-year age range, and 0.15 (logMAR) in children over 6 years old, and at least a two-line BCVA difference between the two eyes (according to the diagnostic criteria for amblyopia established by the Ophthalmology Branch of the Chinese Medical Association in 2011); (3) myopia of no more than −6.00 diopters (D), hypermetropia of no more than + 9.00 D, astigmatism of less than 3.00 D, anisometropia of at least 1.50 D spherical equivalent, or at least 1.00 D cylindrical equivalent; (4) strabismus of no more than 20 prism diopters (Δ) according to prism and alternate cover test (PACT); and (5) better than a 0.7 logMAR BCVA of the amblyopic eye.

*Eligibility exclusion criteria:* (1) ocular diseases, such as ptosis, refractive media opacity, fundus disease, and optic neuropathy; (2) history of interocular or refractive surgery that affects vision; (3) history of treatment for amblyopia in the last 3 months before screening (except spectacle frames); (4) photosensitivity epilepsy; (5) confirmed or suspected conjunctivitis; (6) allergy or intolerance to the test equipment or patch; (7) history of pharmacological intervention that may affect vision such as atropine; (8) participation in another clinical study/trial within 1 month before the enrollment of the study; and (9) failure to comply with optical adaptation.

### Treatment Procedure

**FIGURE 1:**
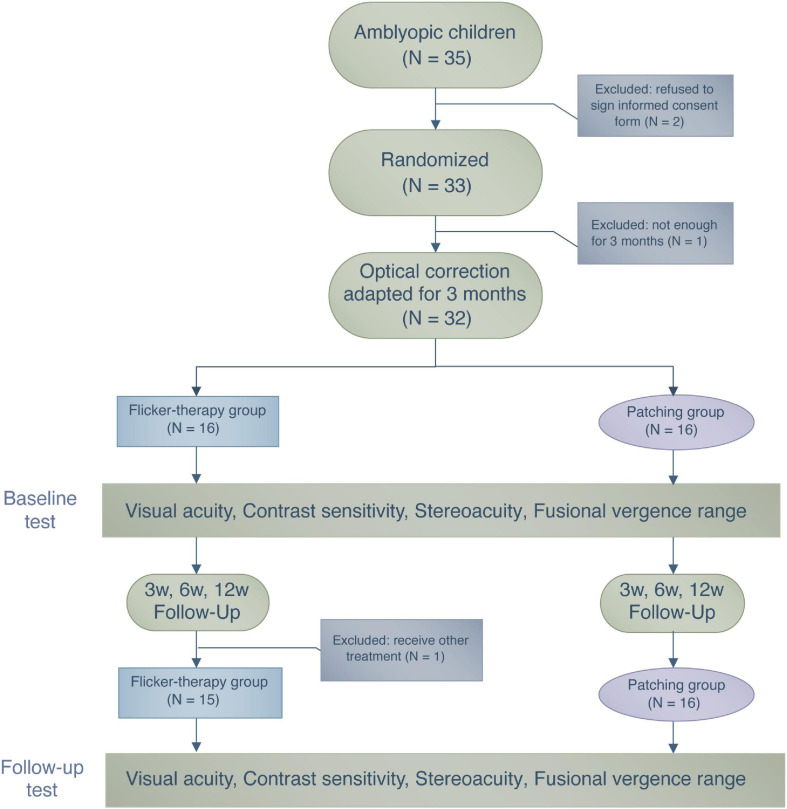
A flowchart illustrating the treatment procedure and the number of patients who participated in this study.

After confirming for the eligibility of each patient, we randomly assigned all participants to one of the two treatment groups using a random number generator so that the group assignment was balanced for both groups (see Figure 1). For all of the patients in Flicker Group and Patching Group, the flicker and patching treatment were combined with optimal refractive correction. As for tracking the compliance, the EFG automatically recorded the duration and time in which the patients wore them. A child has to wear the glasses for 1 h to be counted as complying for the day. On the other hand, we relied on parental diaries, which are not as objective, for measuring daily compliance in the Patching Group. Throughout the study, we asked parents to report for any signs of adverse reactions, such as nausea and double vision, to patching or EFG to our clinic. However, we did not receive any reports of such side-effects.

During the period of wearing the EFG or the patch, patients performed daily activities such as doing homework or watching television. During their first visit (Day 1), guardians of the patients within the Flicker Group were informed about the specifics of the EFG device, such as recharging and handling. Follow-up visits were scheduled after 3, 6, and 12 weeks from their first visit. During each visit, monocular and binocular visual functions were assessed. We had decided to follow up patients for 12 weeks because the investigators who first reported the benefit of the EFG therapy (Vera-Diaz et al., 2016) also followed up their patients for 12 weeks.

#### Flicker Group (Eyetronix Flicker Glass Therapy)

Schor et al. (1976) employed a rate for alternate flicker at 7 Hz, and Vera-Diaz et al. (2016) recently observed a gain in VA of amblyopes with 12 weeks of 7-Hz alternative flicker treatment. Similar to Vera-Diaz et al. (2016), we set the flicker rate of EFG at 7 Hz; the lenses were alternatively transparent (“on”) and opaque (“off”) state for 0.071 + seconds. Also, we set the duty cycle at 50% (50:50 ratio); EFG deprived the normal eye 50% of the time throughout the treatment. Therefore, 1-h treatment with the EFG was equivalent to 30-min deprivation of said eye (50%). The patients were asked to wear the EFG for 1 h/day, 7 days/week throughout the 12-week treatment period.

#### Patching Group (Control)

Patients were instructed to wear a standard, latex-free and adhesive style patch in front of their normal eyes for 2 h, 7 days/week throughout the 12-week treatment period.

#### Visual Acuity

Best-corrected visual acuity was measured separately for each eye using a Tumbling E Logarithmic Visual Acuity Chart (xk100-06, China), which follows the protocol of Early Treatment Diabetic Retinopathy Study. The total score within each line from the Logarithmic Visual Acuity Chart was 0.1 log units. Since there were five letters per line, the correctly read letter was assigned a score of 0.02 log units. The patients were asked to report the orientation of the E letter tested with one eye at 5 m from the chart, and with the non-tested eye occluded throughout the test.

#### Contrast Sensitivity

A CSV-1000 grating chart (VectorVision^®^ Inc., Greenville, OH 45331, United States) is a printed chart-based method to test contrast sensitivity. It presents sinewave targets at four spatial frequencies [3, 6, 12, and 18 cycles per degree (cpd)] at a distance of 40 cm. Subjects were asked to perform a forced-choice task between two targets at different rows: one had a sinusoidal modulation, whereas the other was a mean gray. There are eight levels of contrast (one per column) per spatial frequency. The lowest contrast in the chart is 0.5%. The contrast of patches is presented in decreasing order from left to right. Using the table in the company’s website^2^, we translated the scores (range of 1 to 8) to log units for analysis.

#### Stereopsis

Stereo threshold was measured with a Titmus Stereo test (Stereo Optical Co., Chicago, IL, United States) at a viewing distance of 40 cm under natural light. All patients wore polarizing glasses and, if necessary, additional optical corrections throughout testing. If the patients were not able to perceive the largest disparity given by the Titmus Stereo test, we recorded that the stereoacuity of the patients was “nil” and converted the non-numerical value into 4,500 arcseconds for data analysis.

#### Fusional Vergence Range

Fusional convergence and divergence amplitudes were measured using Synoptophore L-2510B/L-2510HB (Inami & Co., Ltd., Japan). We measured the extent to which the subjects maintained binocular single vision as we increased the vergence demands. Then we computed the total range of fusion, which is the absolute sum of the convergence and divergence break points. The data were recorded in the unit of degrees (deg).

### Data Analysis

Statistical analyses were performed using R software (RStudio, 2016). Data were plotted using Python software (Hunter, 2007). A Shapiro–Wilk test indicated that our dataset of BCVA, contrast sensitivity, and stereopsis did not assume a normal distribution (*p* < 0.05) and failed to meet the requirement for the use of parametric procedures. Therefore, we performed non-parametric (rank-based) analysis of variance (ANOVA)-like computation of longitudinal data using the package “nparLD” designed for R software (Noguchi et al., 2012). *Post hoc* tests were performed with Bonferroni correction when there was a significant main effect of either treatment group or time. We report ANOVA-type statistics (Brunner et al., 2017); 95% confidence intervals (CIs) were obtained from t-distribution approximations. In addition, dataset of fusional vergence range was normally distributed. So we used a parametric two-way mixed ANOVA (within-subject: time, between-subject: group) to compute statistics.

## Results

### Baseline Characteristics

We wanted to ensure that all patients were randomly assigned to the two treatment groups. So we looked for possible differences in baseline between the Flickering and Patching groups via a non-parametric Wilcoxon–Mann–Whitney test. We found that the baseline characteristics, including age, BCVA, contrast sensitivity, stereopsis, fusional vergence range, amblyopic eye’s spherical equivalent, fellow eye’s spherical equivalent, and squint, did not differ between the treatment groups (*p*-values ranged from 0.17 to 0.96) and concluded that the group assignment was random.

### Compliance

We computed compliance by dividing the number of actual days where the patients had undergone the treatment at home from the number of total days for treatment. The mean compliance rate in the Flicker Group was 93.80 ± 0.025%, whereas the mean compliance rate in the Patching Group was 93.97 ± 0.021%. A non-parametric Wilcoxon–Mann–Whitney test revealed no significant difference in compliance rate between the two treatment groups (*W* = 116, *p* = 0.89).

### Comparison Between the Eyetronix Flicker Glass Therapy and the Standard Patching Therapy

#### Improvement of Best-Corrected Visual Acuity (Primary Outcome)

We firstly conducted a non-parametric mixed ANOVA-like test (see *Materials and Methods*), which includes between-subject factor (treatment group) and within-subject factor (time). It revealed no main effect of group (*F*_(__1_._00_,_∞__)_ = 1.57, *p* = 0.21) but a significant effect of time (*F*_(__2_._37_,_∞__)_ = 40.42, *p* < 0.001). Then we performed a *post hoc* analysis for each pairwise comparison (between two timepoints per group) with Bonferroni correction. We found a significant difference between baseline and 12 weeks in Flicker Group (*p* < 0.001, Cohen’s *d* = 0.86, 95% CI: [0.016, 0.24] logMAR). We also found a significant difference between baseline and 12 weeks in the Patching Group (*p* < 0.001, Cohen’s *d* = 1.08, 95% CI: [0.068, 0.34] logMAR). These results show that both groups improved BCVA significantly over 12 weeks. However, according to the ANOVA test, there was no significant difference in VA improvement at 12 weeks relative to baseline between the two groups (*F*_(__1_._00_,_∞__)_ = 2.26, *p* = 0.13). In particular, the mean improvement (mean ± SD) in BCVA at 12 weeks relative to baseline in the Patching Group (0.21 ± 0.14 logMAR) was slightly (but not significantly) higher than in the Flicker Group (0.13 ± 0.11 logMAR).

**FIGURE 2:**
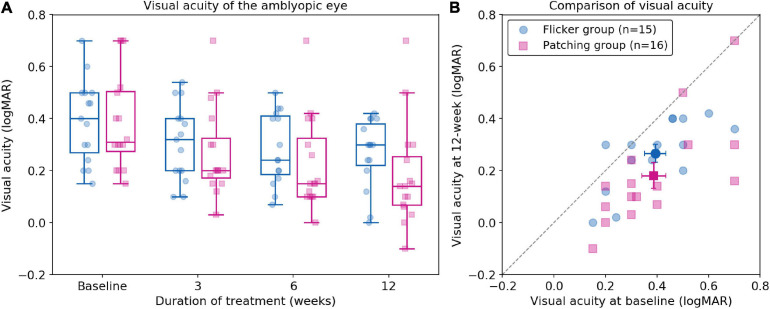
Best-corrected visual acuity following treatment. **(A)** Boxplots of best correct visual acuity (BCVA) of the amblyopic eye (in logMAR) in Flicker and Patching treatment groups. Blue represents the Flicker Group, and pink the Patching Group. Individual data point is represented by a dot. The black solid line within each box represents the median. The box represents the interquartile range (IQR) of the data (25th to 75th percentile). The whisker represents 1.5 × IQR either above the third quartile or below the first quartile. **(B)** Individual data point of visual acuity (BCVA) in patients’ amblyopic eyes measured at baseline and at 12 weeks of treatment. The dashed line represents unity. Blue circles represent the Flicker Group, whereas pink triangles represent the Patching Group. Points below the dashed line (unity line) show an improved BCVA at week 12 relative to baseline.

Also, we found a significant interaction between the treatment group and time (*F*_(__2_._29_,_∞__)_ = 4.39, *p* = 0.0091). This indicates that the rate of BCVA improvement was significantly faster in the Patching Group than in the Flicker Group. Figure 2B shows that most points are under the dashed line (unity line); this indicates that most children in both groups experienced an increase in BCVA after 12 weeks of treatment.

Interestingly, we observed a significant negative correlation between baseline VA and VA improvement at 12 weeks in the Flicker Group (*r* = −0.56, *p* = 0.029). This indicates that the worse the baseline VA, the larger the VA improvement at 12 weeks. However, we did not find a significant correlation in the Patching Group. We also examined the relationship between age and VA improvement at 12 weeks. According to a Pearson correlation test, we found no significant relationship in both Flicker (rho = −0.20, *p* = 0.48) and Patching (rho = 0.14, *p* = 0.60) groups.

#### Contrast Sensitivity

**FIGURE 3:**
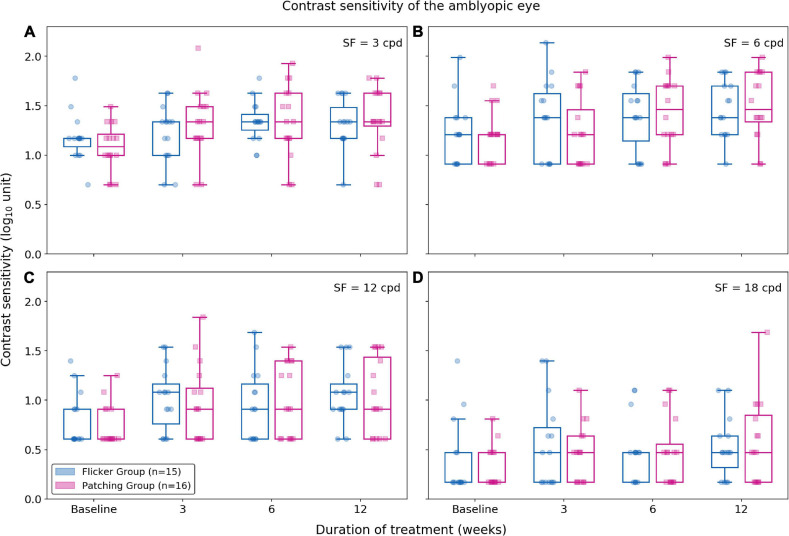
Contrast sensitivity at 3, 6, 12, and 18 cpd (cycles per degree) for each treatment group. Blue plots represent the Flicker Group, and pink plots the Patching Group. Circles represent the contrast sensitivity of patients in the Flicker Group, and squares the contrast sensitivity patients in the Patching Group. **(A)** Contrast sensitivity for 3 cpd. **(B)** Contrast sensitivity for 6 cpd. **(C)** Contrast sensitivity for 12 cpd. **(D)** Contrast sensitivity for 18 cpd.

Using the non-parametric ANOVA test (within-subject factor: time, between-subject factor: group), we found no significance difference between Flicker and Patching groups (*p*’s < 0.05; see Figure 3). However, we found a significant effect of time at all spatial frequencies (*p*’s < 0.001). So we compared baseline and 12 weeks as *post hoc* analysis at each spatial frequency and treatment group. In the Flicker Group, we observed a significance improvement (*p*’s < 0.05) at 12-week relative to baseline for contrast sensitivity at 3 and 12 cpd but not at 6 and 18 cpd. The mean improvements (mean ± SD) for 3, 6, 12, and 18 cpd between baseline and 12 weeks were 0.14 ± 0.271, 0.23 ± 0.43, 0.28 ± 0.40, and 0.13 ± 0.52 respectively. For the Patching Group, there was a significance improvement (*p*’s < 0.05) at 12-week relative to baseline for 3, 6, and 12 cpd but not at 18 cpd. This means that contrast sensitivity for 3, 6, and 12 cpd improved significantly throughout the treatment. The mean improvements (mean ± SD) for 3, 6, 12, and 18 cpd between baseline and 12 weeks were 0.28 ± 0.46, 0.31 ± 0.33, 0.30 ± 0.44, and 0.25 ± 0.47, respectively.

For all spatial frequencies, we also evaluated the relationship between age and improvement in contrast sensitivity using a Pearson correlation test. For the Flicker Group, there was no correlation between age and improvement (*p*’s > 0.05). For the Patching Group, there was no significant correlation (*p*’s > 0.05) except at 3 cpd (rho = 0.58, *p* = 0.018).

#### Stereopsis

**FIGURE 4:**
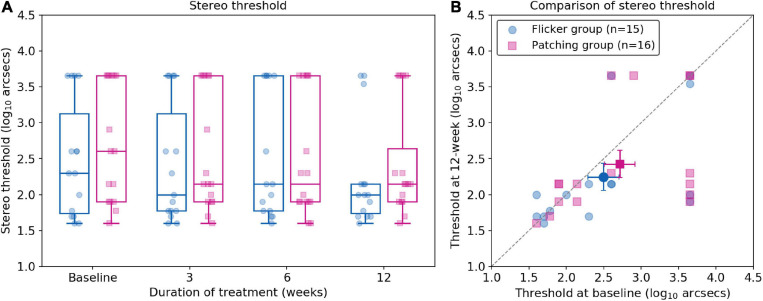
Stereo thresholds (log arcsecs) as measured with the Titmus Stereo test. **(A)** Boxplots of stereo thresholds in Flicker and Patching treatment groups. **(B)** Individual data point of stereo thresholds measured at baseline and at 12 weeks of treatment. Both panels are plotted similarly to Figure 2.

Non-parametric mixed ANOVA-like test revealed no main effect of group (*F*_(__1_._00_,_∞__)_ = 0.52, *p* = 0.47) and no significant effect of time (*F*_(__2_._46_,_∞__)_ = 2.18, *p* = 0.10). Also, it revealed no interaction effect between the treatment group and time (*F*_(__2_._46_,_∞__)_ = 0.56, *p* = 0.61). For the Flicker Group, the mean improvement in stereo threshold was 0.573 ± 1.62 log_10_ arcsecs (mean ± SD) at 12 weeks. For the Patching Group, the mean improvement in stereo threshold was 0.662 ± 1.93 log_10_ arcsecs (mean ± SD) at 12 weeks. Both improvements were not significant (*p*’s > 0.05) relative to baseline (see Figure 4).

#### Fusional Vergence Range

**FIGURE 5:**
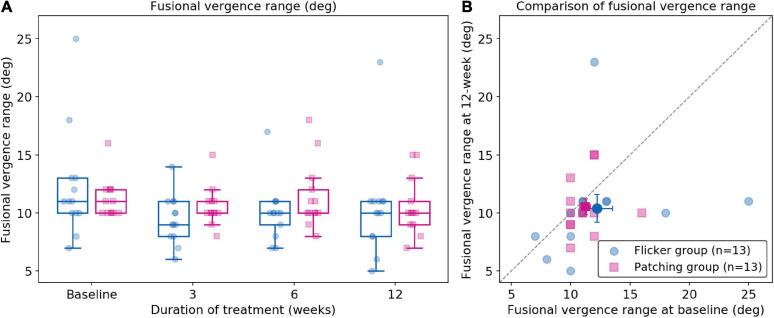
Fusional vergence range (deg). **(A)** Fusional vergence range in Flicker and Patching treatment groups. **(B)** Individual data point of fusional vergence range measured at baseline and at 12 weeks of treatment. Both panels are plotted similarly to Figure 2.

Five patients (two in the Flicker Group and three in the Patching Group) were not able to complete the test of fusional vergence range and therefore had to be excluded in data analysis (see Figure 5). A two-way mixed ANOVA showed no significant effect of group (*F*_(__1_,_24__)_ = 0.24, *p* = 0.63) and no significant effect of time (*F*_(__3_,_72__)_ = 2.19, *p* = 0.096). It revealed no significant interaction effect between the treatment group and time (*F*_(__3_,_72__)_ = 0.77, *p* = 0.34). In short, for both groups, no significant difference was observed between baseline and 12 weeks. However, the mean changes in fusional vergence range for both groups slightly deteriorated. In the Flicker Group, the mean change (mean ± SD) was −1.85 ± 5.63 deg, whereas in the Patching Group, the mean change was −0.692 ± 2.75 deg.

## Discussion

Our results showed that the EFG therapy is not superior to patching for amblyopia in children. The gain in VA between baseline and 12 weeks was slightly larger in patching (0.21 ± 0.14 logMAR) than EFG therapy (0.13 ± 0.11 logMAR). The VA gain in EFG therapy is similar to what a previous EFG study reports at 12 weeks (0.12 ± 0.11 logMAR) (Vera-Diaz et al., 2016). However, the difference in VA gain between the EFG and patching therapies was not statistically significant. In addition, both therapies induced a significant improvement in VA at 12 weeks relative to baseline. Also, both treatment groups showed a gain in contrast sensitivity at 3 and 12 cpd; however, only patching improved the contrast sensitivity at 6 cpd. Stereo threshold and fusional vergence range did not significantly improve in both groups.

The gain in VA from 12-week EFG therapy (0.13 logMAR) reported in our study is similar to that from binocular therapies. For instance, a pediatric study on dichoptic perceptual learning in 14 amblyopic children shows a VA gain of 0.1 logMAR (Knox et al., 2012). However, the dichoptic therapy seems to be more effective than the EFG therapy because the children completed five training sessions (1 h each) total in 1 week rather than undergoing a daily 1-h treatment for 12 weeks (i.e., our EFG therapy). Moreover, studies show that a binocular iPad therapy improves VA by 0.08–0.09 logMAR at 4 weeks (Li et al., 2014; Birch et al., 2015; Birch et al., 2020) and 0.105 logMAR at 16 weeks (Holmes et al., 2016). These improvements are similar to what we found at 3 (0.085 logMAR) and 12 weeks (0.13 logMAR) of EFG treatment. However, other RCTs show that a binocular iPad therapy is more effective than an EFG therapy. They report a VA gain of 0.15 logMAR after 2 to 8 weeks of a binocular iPad therapy (Li et al., 2015a; Kelly et al., 2016). An EFG therapy seems to be superior I-BiT therapy, which preferentially stimulates the amblyopic eye without depriving the input to the fellow eye. To illustrate, an RCT shows that 3 h of weekly treatment using I-BiT reports a gain of 0.07 logMAR in the amblyopic eye at 6 weeks (Foss et al., 2013), whereas we report a gain of 0.11 logMAR at 6 weeks. To evaluate whether an EFG therapy stands in all of these treatment options, a future RCT should test a larger pool of patients and compare the therapies.

We found that the rate of VA improvement in the Patching Group was significantly faster than in the Flicker Group. It seems that the VA gain did not plateau earlier in the Patching Group, since the improvement relative to baseline was larger at 12 weeks (0.21 ± 0.14 logMAR) than 6 weeks (0.16 ± 0.10 logMAR). Therefore, it seems that the VA improvements in EFG therapy were smaller in comparison with patching. There could be a few possible explanations for this difference. First, patching might simply be slightly superior for improving VA to EFG therapy. To illustrate, the age distribution of the patients for the Patching Group (6.38 ± 2.45 years, mean ± SD) was slightly (but not significantly) different than that in the Flicker Group (5.27 ± 1.10 years, mean ± SD). The former group had four more children in the age between 8 and 13 years. Studies have shown that there is an age-dependent response to patching in favor of younger patients (Fronius et al., 2014). Therefore, the rate of VA improvement from patching would have been even faster if the age distribution between the two groups was more similar. Second, the treatment durations of the two therapies differed. We acknowledge that the shorter duration of EFG therapy (i.e., 1 h/day) might have contributed to the slower rate of VA gain than that in patching (2 h/day). To our limited knowledge, there is no direct evidence to show that a longer daily duration of EFG treatment produces more VA gain. However, a patching therapy study reports that dose rates of 2 to 6 h/day produced the same visual outcome, although the rate of visual gain was quicker in a larger dose of patching treatment (Stewart et al., 2004). We had decided to administer EFG therapy for 1 h/day, rather than 2 h/day, for two reasons: 1) Eyetronix^®^ claims that daily dose of 1–2 h is sufficient^3^, and (2) the battery life of our EFG lasted for only 1.5 h. Despite the difference in treatment duration between the two groups, we did not find a significant difference in VA improvement at 12 weeks.

Eyetronix^®^ categorizes the EFG therapy as binocular because a dichoptic flicker has been shown to affect interocular interaction (Schor et al., 1976). To illustrate, using a binocular VA test, Schor et al. (1976) showed that the influence of the fellow eye on amblyopic eye’s perception was minimized while visual targets were alternatively presented to the eyes at 7 Hz (i.e., dichoptic flicker) (Schor et al., 1976). Another study shows that binocular (but not dichoptic) flicker can affect binocular interaction (Kosovicheva et al., 2019). It claims that EFG can “break suppression and restore normal binocular fusion” (See Text Footnote 3). However, the categorization is open to debate. Unlike EFG, which deprives monocular input at all times, binocular therapies show stimuli to both eyes simultaneously. For example, a binocular therapy, be it movie viewing (Li et al., 2015b) or game playing (Knox et al., 2012), displays stimuli to both eyes simultaneously. Moreover, we do not see an improvement in stereopsis and fusional vergence range as some studies of binocular therapies have shown. For instance, dichoptic anti-suppression therapies, which reduce the suppression by lowering the contrast for the fellow eye, have been reported to bring binocular benefits (Hess et al., 2010; Li et al., 2013; Vedamurthy et al., 2015). Moreover, a laboratory study reports that a dichoptic virtual reality display, which does not reduce the contrast for the fellow eye, reduces suppression in normal adults (Bao et al., 2017). This dichoptic design is analogous to the EFG therapy, which deprives each eye equally without rebalancing the contrast. It is very likely that these different designs of binocular treatment could be accounted for by different mechanisms.

It should be noted that the reported interocular suppression changes were measured while subjects were viewing with dichoptic flicker (Schor et al., 1976). Would there be the same effect when patients are no longer viewing with the flicker? We do not think so because we did not observe binocular benefits after EFG treatment. However, our results disagree with a previous report from Eyetronix^®^ (Vera-Diaz et al., 2016), which shows an improvement in stereopsis. This might be accounted for by the different doses of treatment in each day (2 vs. 1 h) or different stereo measures (Random Dot 2 test vs. Titmus). Nevertheless, one would wonder why we had not measured suppression in our RCT. We had not done so because children have found psychophysical tasks (i.e., dichoptic motion coherence task) that measure suppression challenging (Li et al., 2014; Li et al., 2015b). We are currently working on a study to investigate the effect of the EFG therapy on suppression in adults.

Binocular therapies of amblyopia, such as dichoptic training, perceptual learning, and video gaming, show promise for treating adults (Li et al., 2013; Hess and Thompson, 2015; Vedamurthy et al., 2016). On the other hand, monocular therapies except refractive therapy (Gao et al., 2018) show that younger patients benefit more than older ones (Stewart et al., 2007; Fronius et al., 2014). Therefore, whether EFG therapy, which is effective in improving amblyopic children’s VA, is also effective for amblyopic adults should also be explored in the future.

### Limitations

Our study has several limitations. First, we did not have a large sample size. Second, the durations of the two therapies were different. Third, we did not perform follow-up assessments of the patients after the end of the treatment. Therefore, whether the monocular benefits from the EFG therapy last remains to be investigated.

## Conclusion

An EFG therapy and patching brought a similar VA gain for children with amblyopia at 12-week visit. Both therapies resulted in a gain in contrast sensitivity at 3 and 12 cpd; however, only patching improved the contrast sensitivity at 6 cpd. Both therapies did not benefit binocular visual functions (stereopsis and fusional vergence range). We believe that EFG can be an additional choice for therapy.

## Data Availability Statement

The raw data supporting the conclusions of this article will be made available by the authors, without undue reservation.

## Ethics Statement

The studies involving human participants were reviewed and approved by Ethics Committee of the Affiliated Eye Hospital at Wenzhou Medical University. Written informed consent to participate in this study was provided by the participants’ legal guardian/next of kin.

## Author Contributions

XY, JX, YW, and JZ conceived the experiments. BC and HC performed the experiments. SM, SC, and JZ analyzed and interpreted the data, and wrote the manuscript. All authors contributed to manuscript revision, read and approved the submitted version.

## Conflict of Interest

The authors declare that the research was conducted in the absence of any commercial or financial relationships that could be construed as a potential conflict of interest.

## Footnotes

1 http://www.chictr.org.cn

2 http://www.vectorvision.com/csv1000-norms/

3 http://eyetronix.com/
